# A Rubik’s microfluidic cube

**DOI:** 10.1038/s41378-020-0136-4

**Published:** 2020-06-15

**Authors:** Xiaochen Lai, Zhi Shi, Zhihua Pu, Penghao Zhang, Xingguo Zhang, Haixia Yu, Dachao Li

**Affiliations:** 1 State Key Laboratory of Precision Measurement Technology and Instruments, Tianjin University, Tianjin, 300072 China; 2 Tianjin Key Laboratory of Biomedical Detecting Techniques and Instruments, Tianjin University, Tianjin, 300072 China

**Keywords:** Engineering, Physics

## Abstract

A Rubik’s cube as a reconfigurable microfluidic system is presented in this work. Composed of physically interlocking microfluidic blocks, the microfluidic cube enables the on-site design and configuration of custom microfluidics by twisting the faces of the cube. The reconfiguration of the microfluidics could be done by solving an ordinary Rubik’s cube with the help of Rubik’s cube algorithms and computer programs. An O-ring-aided strategy is used to enable self-sealing and the automatic alignment of the microfluidic cube blocks. Owing to the interlocking mechanics of cube blocks, the proposed microfluidic cube exhibits good reconfigurability and robustness in versatile applications and proves to be a promising candidate for the rapid deployment of microfluidic systems in resource-limited settings.

## Solving the puzzle of modular microfluidics

A modular system modeled on the Rubik’s Cube could give researchers a versatile framework for designing and reconfiguring microfluidic systems. Many groups have pursued microfluidic building blocks that can be arranged in diverse conformations depending on the needs of the experiment. Dachao Li and colleagues at Tianjin University in China have drawn inspiration from a popular puzzle toy to develop a 3D-printed microfluidic system that can be easily twisted and turned to change its function. They mimicked the Rubik’s Cube design with modular pieces that contain distinct microchannel layouts, flanked with O-rings that achieve a tight leakproof seal regardless of how the device is arranged. Li’s team use a single device to perform fluid mixing and droplet-based microbial culture, and foresee the potential to introduce diverse functions including pumps, sensors, and valves in future iterations.

## Graphical abstract

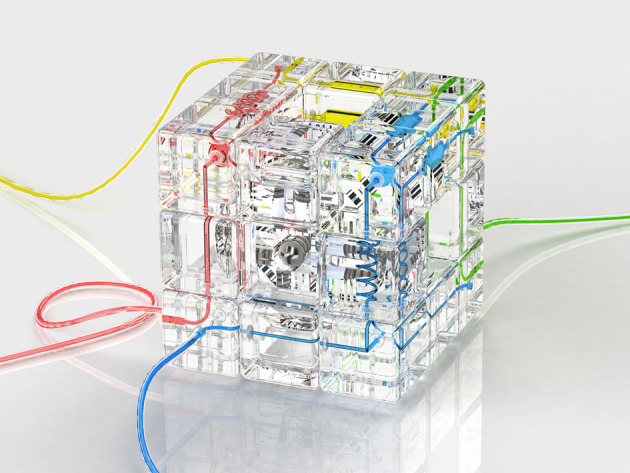

## Introduction

The usefulness of microfluidics cannot be overestimated in today’s scientific research. Apart from the original use in chemical analysis^1^, the unmatched advantages of microfluidics, such as low consumption of reagents, fast reaction speed, and high throughput^2^, have brought forth endless possibilities in a large range of subjects, such as chemical synthesis^3^, materials science^4,5^, biology^6^, and clinical diagnosis^7^. Nevertheless, microfluidics technology is in its development stage, and the potential of microfluidics has yet to be fully exploited. That is, there is an increasing demand for customized microfluidic systems featuring various applications. However, fabricating a custom microfluidic chip can be expensive, laborious, and very time consuming. Although the emergence of soft lithography techniques and the use of elastomers have greatly simplified fabrication^8^, such processes are still highly dependent on professional facilities and expert operators and therefore remain unreachable to many unequipped laboratories, not to mention the on-site deployment of microfluidics in resource-limited settings.

In recent years, the microfluidic community has witnessed the rapid development of novel fabrication techniques^9,10^ that are suited for the simplified customization of microfluidic systems. Among these, three-dimensional (3D) printing, the most representative approach known for its straightforward manner^11,12^, has been used to directly create arbitrary microfluidic structures. State-of-art 3D printing techniques have achieved channel cross-sections as small as 18 μm × 20 μm using desktop 3D printers^13^ and even submicron scale microfluidic structures by two-photon polymerization^14^, which are fine enough for most microfluidic applications. However, 3D printing can only yield monolithic devices, and the design of microfluidics must be done during the prefabrication stage. In applications such as device prototyping and point-of-care testing, where the rapid on-site customization and modification of the microfluidics platform are desirable^15^, 3D printing becomes inefficient due to its lengthy cycle time from design to use.

To enable the rapid deployment of customized microfluidic systems, the concept of “modular microfluidics” is proposed^16–25^. In modular microfluidics, individual microfluidic blocks are created in a modular design and assembled to form a system. Owing to this flexible design, modular microfluidics allows the design and reconfiguration of the microfluidics system during the postfabrication stage. In previous studies, microfluidic blocks were created in the form of jigsaw puzzle-like blocks^16,17^, Lego-like blocks^18,24,25^, magnetic blocks^22^, and other designs^19,23^ to allow the versatile combination of different components. The modular microfluidics concept exhibits good adaptability in various applications and have become a promising approach for rapid on-site customization. Nevertheless, modular microfluidics has limitations when compared with monolithic microfluidics. The most common but critical problem is “leakage.” Owing to the unstable connections between discrete element blocks, fluids tend to leak under the effect of high pressure. In some cases, additional mechanical components such as metal connection pins^19^ and screws^23^ have been used to achieve higher pressure tolerance, but these components have also increased the complexity of the reconfiguration process and limited the usability of the system. It would be desirable to find a solution towards modular microfluidics that has both excellent performance and good usability so that it could fulfill the requirements for reliable and highly customizable microfluidic systems in resource-limited settings.

In this work, we proposed a reconfigurable microfluidic system adapted from Rubik’s cube, a 3D combination puzzle game that has gained worldwide popularity over the past 40 years. There are several unique features of Rubik’s cube that make it a good candidate for modular microfluidics. First, Rubik’s cube has an ingenious interlocking mechanism for its components so that every block is firmly attached to its neighboring blocks, which could resolve the leakage issue while ensuring an easy reconfiguration process. Second, the permutation of the cube from one state to another only requires a maximum of 20 twists of the cube, and the rules could be learned by even preschool children, which ensures an easy-to-operate system for end users. Finally, there are over 4.3 × 10^19^ possible combinations for a 3 × 3 × 3 Rubik’s cube,^26^ that is, the cube can be scrambled into numerous states from the same starting position, so diverse possibilities of microfluidic configurations are available.

With reference to the original design of Rubik’s cube, we created and tested a Rubik cube-based microfluidic system. Unlike previously reported modular microfluidics that use extra reinforcing accessories to prevent leakage, the microfluidic cube enabled high pressure tolerance without requiring extra measures after reconfiguration. Moreover, when reconfiguring the microfluidics, the cube-like system does not require dissembling and reassembling the whole system; instead, it only needs a few rotations, which means a faster and more convenient reconfiguration process. In addition, the microfluidic cube exhibited ease of use and robustness during the repeated reconfiguration processes. Considering its advantages in performance, convenience, and ease of use, the proposed Rubik’s cube-like microfluidic system could provide an easy and affordable solution to the rapid deployment of microfluidics, which could pave the way towards highly customized applications in resource-limited settings.

## Results

### Design of the microfluidic cube

**Fig. 1:**
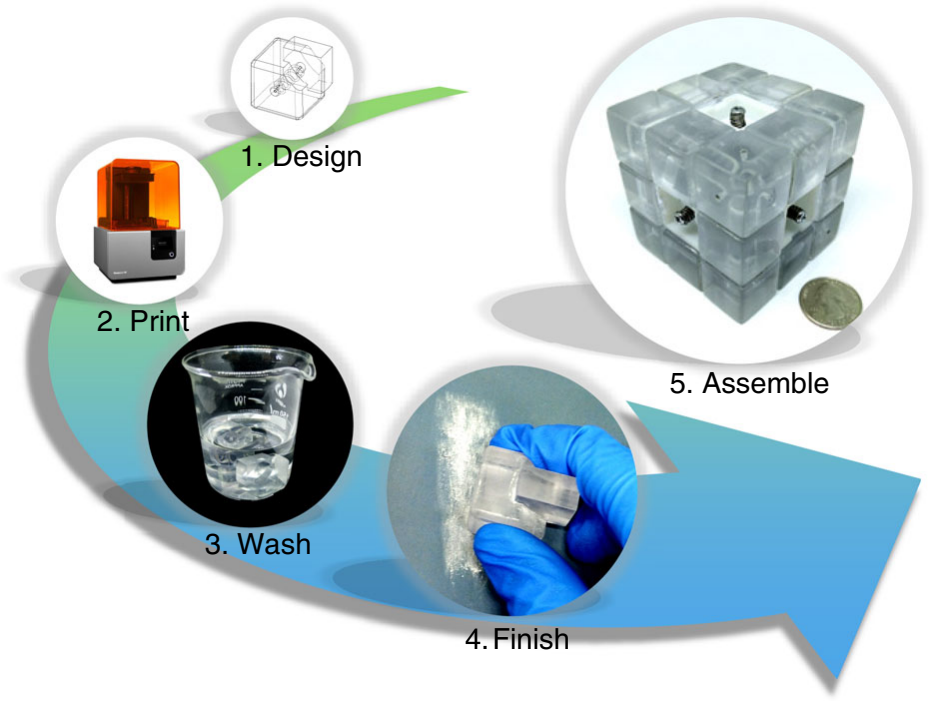
Fabrication process of a microfluidic cube

**Fig. 2:**
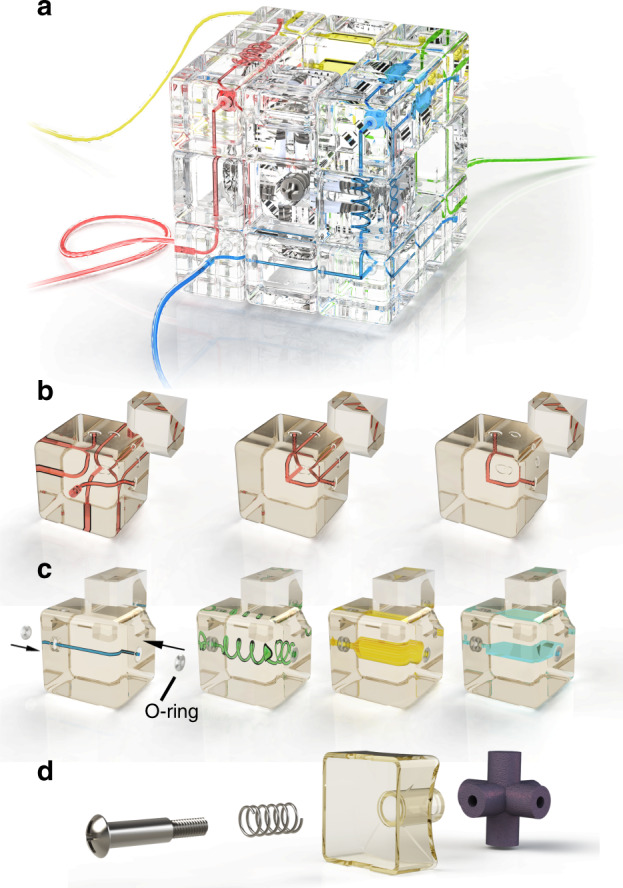
Illustration of the proposed Rubik’s cube-like microfluidic system. **a** Overall illustration of the cube. **b** Corner blocks of the microfluidic cube, including three-way inlets/outlets (left), 3D T-junction(middle). and turning (right). **c** Edge blocks of the microfluidic cube, from left to right are the straight channel, spiral channel, 3D chamber, and planar chamber, respectively. **d** Central block and other components of the cube

Based on the original design of Rubik’s cube, we proposed a “Rubik’s microfluidic cube,” where microfluidic components are built in Rubik’s cube, and the reconfiguration of microfluidics could be accomplished by playing the cube. The fabrication process of the microfluidic cube is illustrated in Fig. 1, and detailed information is described in the Materials and methods section. Figure 2a shows the overall design of the proposed Rubik’s cube-like microfluidic system. The system looks like an ordinary Rubik’s cube, but all 12 edge cubes and 8 corner cubes are replaced with blocks containing internal microchannels. The edge blocks are blocks on the center of each of the 12 edges of the cube, which are designed as channels and chambers that perform microfluidic functions. The corner blocks, which are located on the eight corners of the cube, are mainly used as junctions and in/outlets. The central blocks on the center of each of the six faces of the cube have no internal void structure, but are essential in maintaining the cube’s integrity. Figure 2b, c show some basic designs of the edge and corner blocks used in the microfluidic cube, respectively. Each of the edge and corner blocks is an independent microfluidic chip, with its inlet/outlet located at the geometrical center of a surface. All these blocks were 3D printed using a desktop stereolithography (SLA) printer. Clear resin was used to obtain transparent blocks for ease of observation. In addition, two silicone rubber O-rings are embedded into each edge block. Figure 2d shows an enlarged view of how a central block is fixed onto the cube core. Apart from the 3D-printed core and central blocks, springs and screws are used to push the central block to the core. After all blocks are assembled, the pushing force from the spring acting on the central blocks will be dispersed to all blocks due to the interlocking mechanism, ensuring an integrated system while allowing the smooth rotation of the cube faces.

**Fig. 3:**
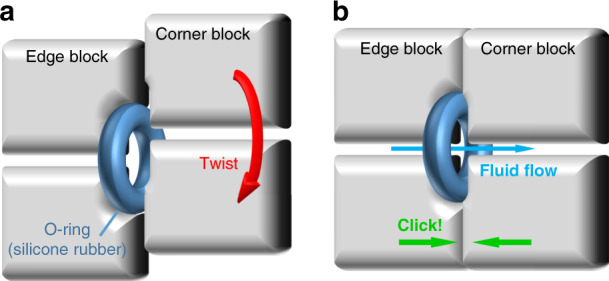
Cross-section illustration of the O-ring guided alignment and leakproofing at the end of a rotation. **a** When the corner block is not rotated to the right position, a gap exists between two blocks that will cause leakage. **b** When the corner block is rotated to the right position, the O-ring embedded into the edge block will automatically fit into the concave on corner blocks, ensuring a self-aligned and leakproof connection of two blocks

As 3D printing produces rigid blocks with imperfect features (deformation due to resin shrinking and artificial defects caused by the washing/finishing process), in most situations, the contacting surfaces of neighboring blocks cannot perfectly fit to each other, and leakage can occur if no further measures are taken. To address the leakage issue, an O-ring-aided sealing strategy is employed, as shown in Fig. 3. On the contacting surfaces, the edge blocks have a deep torus concave around the fluid channel, and a silicone rubber O-ring is tightly embedded into the concave, leaving a small portion unburied. On the corner blocks, there is also a torus concave structure that is shallow in depth. In the process of twisting a face of the cube, there should be a gap between the edge and corner blocks (Fig. 3a). However, when the corner block is twisted to the aligned position, the O-ring in the edge block will automatically fit into the concave structure on the corner block (Fig. 3b), ensuring a sealed contact between the blocks. Moreover, the O-ring fitted into the corner block will prevent further movement of the corner block after a complete rotation, enabling the automated alignment of the blocks.

### Characterization of the microfluidic cube

After the fabrication and assembly of the cube blocks, the cube was tested to evaluate its performance. Two of the key factors that determine the performance of the microfluidic cube are the dimension and tolerance of the cube blocks, as the fabrication error could determine the precise alignment of cube blocks. We measured the internal and external dimensions of the microfluidic blocks in comparison with their designed value, as shown in Table 1. The result reveals that the dimensions of the final blocks could vary from our original design, where the internal dimensions (channels) are smaller than designed and the external dimensions are larger than designed. The large fabrication error of the 3D-printed components derives from several factors: the resin shrinkage throughout the printing process, the laser spot size, and the printing parameters. However, although discrepancies exist between the designed and final cube blocks, the actual devices of the same dimension showed relatively uniform deviation since the abovementioned factors have similar effects on the devices fabricated. Moreover, we find that the fabrication error of blocks did not cause leakage of the fluid when the microfluidic cube was working. This is mainly because the misalignment of blocks could be compensated with the help of the O-ring-aided sealing strategy. Because the silicone rubber O-ring is flexible and could deform to fit the geometries of the distorted blocks and prevent leakage, it enables the sealing of channels on adjacent blocks that have insignificant discrepancies. In conclusion, although the fabricated microfluidic blocks could have deformed geometries from their original design, the final device is able to align with each other to give a sealed and functioning microfluidic configuration. In the future, it is anticipated that more advanced 3D printing technologies could build more precisely defined blocks to enable the perfect alignment of contacting blocks.

**Table 1:** Comparison of dimensions of the designed and fabricated blocks

	Channel width (μm)				External dimensions (mm)	
Designed	500.00	800.00	1000.00	2000.00	21.50	9.00
Measured (*n* = 5)	395.65	674.40	925.56	1928.74	21.65	9.10
SD	60.17	34.11	28.30	40.88	0.05	0.03

The pressure resistance is another essential consideration for evaluating the performance of microfluidics systems. Here, we studied the pressure resistance of the microfluidic cube. The pressure resistance should depend on the tightness of the spring on the central blocks since the spring provides the pushing force that keeps the cube blocks together. In our assembly, the screws are driven 3.5 mm into the cube core to tighten the screw, as illustrated in Supplementary Fig. S1. In this case, the pushing force from the spring is moderate, allowing both smooth rotation of the cube and leakproof fluid flow. The configuration for the pressure resistance test is shown in Supplementary Fig. S2, where the channel is filled with water at first, and then a turning corner as a dead-end block replaces the outlet to shut out flux and maintain the internal pressure. No liquid leakage is observed during the process of increasing the inlet pressure from 0 to 5 bar, indicating an excellent pressure resistance that is sufficient for most microfluidic applications. Such high pressure resistance could be explained by the structure of the cube; when turned to an aligned position, the edges are in contact with corners only at the silicone O-ring, giving a very small contact area. As a result, the pushing force from the spring is totally dispersed on the silicone O-rings, providing a large local pressure at the O-ring/corner block interface, resulting in a very large pressure resistance.

In using the microfluidic cube, the observation of the microchannels could be different from ordinary microfluidic devices, since the system size and distance from channel to surface is relatively larger than the usual approaches. In this work, we could observe the microchannels on the top face of the cube using a stereomicroscope or the side faces using a desktop USB magnifier, as depicted in Supplementary Fig. S3. The quality of the image is acceptable for general applications. However, considering the distance between the channel and cube surface and the overall size of the cube, it could be a little challenging to focus on the channel using an ordinary microscope, especially in the situation of capturing a high magnification image. To address this issue, we could build specific blocks with biased channels/chambers near the surface (but the in/outlets remain at the center of the blocks for alignment with other blocks) for high-quality imaging. In the future, we also plan to add custom observation blocks that are integrated with lens and camera modules for more convenient and self-sufficient observation of the microchannels^27^.

### Reconfiguring the microfluidic cube

**Fig. 4:**
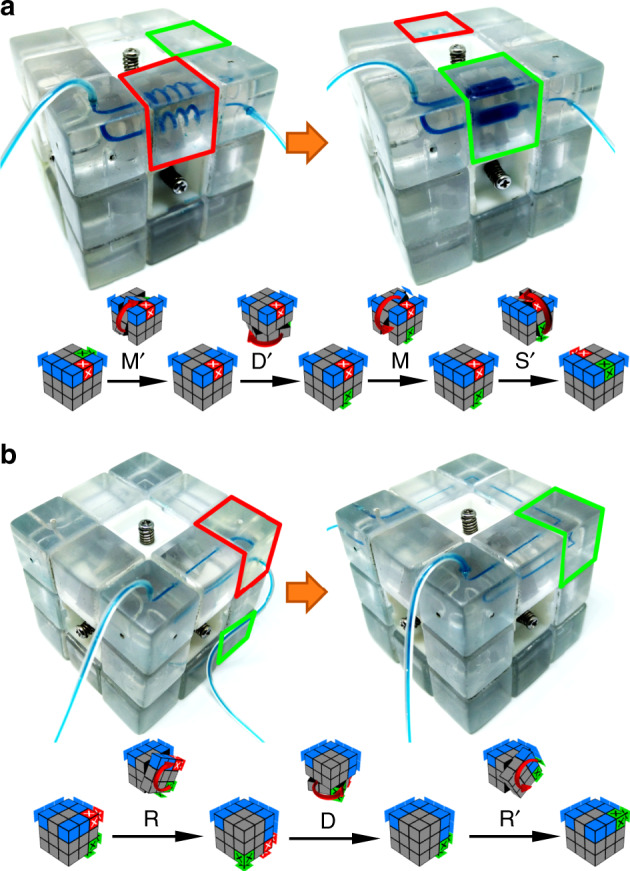
Turning a specific edge/corner block to the desired position. **a** Replacing an edge block in the fluid path. The algorithm for this change: M′D′MS′. **b** Replacing a corner block in the fluid path. The algorithm for this change: RDR′. The bottom illustrations represent the twisting procedure, where we wish to substitute the red block with the green block while keeping the blue blocks unchanged. Explanations of the notations can be found in ref. ^26^

The reconfiguration of microfluidics could be performed by turning the faces of the cube. However, how can one determine the faces, directions, and sequence of turns to achieve the desired microfluidic system? This can be achieved by following Rubik’s algorithms. Rubik’s algorithms are sets of memorized moves that have a specific effect on the cube. The sequence of movements of an algorithm is usually referred to as Singmaster notation^28^, where capital letters are used to represent each move. The desired effect of algorithms, such as replacing one block with another, is useful in the customization of microfluidics. For instance, Fig. 4a shows the process of substituting an edge block (red) in the fluid circuit with another (green) while keeping other useful blocks (blue) unchanged. The process can be performed using an algorithm with four steps. Similarly, Fig. 4b shows the three-step process of substituting a corner block (red) with another (green) while keeping the desired blocks (blue) unchanged. To help the readers understand the algorithm, vivid demonstrations of the switching of the cube blocks are also available in Supplementary Movies S1 and S2. For those who are familiar with Rubik’s cube, such transformations can be performed in a few seconds. Notably, apart from the desired effects, many algorithms have side effects from changing other parts of the cube. Such algorithms, including the two examples in Fig. 4, usually have fewer moves than algorithms that have fewer side effects. In most cases, where only a few blocks of the cube are utilized and the other parts of the system are insensitive to unexpected changes, these simpler algorithms can be used for faster transformation.

With the help of algorithms, we can designate the position of most of the blocks in the cube to customize the microfluidics. However, it is not always possible to designate the position and orientation of every block in the cube. This is because of the intrinsic limitations of Rubik’s cube: for legal moves, (1) the orientation of the last edge and corner blocks is automatically determined, and (2) all pieces must be in an even permutation^29^. For easy understanding, imagine a standard Rubik’s cube. If Rubik’s cube is disassembled and then reassembled randomly, there is only 1/12 chance that the cube can be completely restored. Attempts to restore such a cube could encounter situations that cannot be dealt with at the final steps, such as wrong orientation and piece swaps of the last few cubes^30^. Similarly, if we regard the microfluidic cube with all pieces at designated positions as the unscrambled state of the cube, then there is a chance that the cube cannot be restored to that state. However, in most cases, we do not need to worry about the solvability problem. This is because (1) most of the pieces in the microfluidic cube are symmetric and free from orientation limitation; and (2) the cube is always partially solvable, except for the last few blocks. For most microfluidic systems that do not utilize all 20 fluidic blocks in the cube, the unused blocks can be designated problematic blocks so that an algorithm for that state can be developed.

**Fig. 5:**
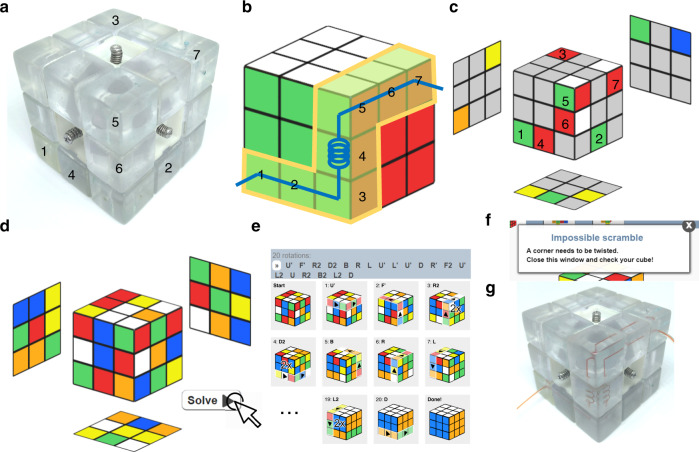
Finding and applying the optimized algorithm for the customization of microfluidics using an online Rubik’s solver. **a** Observe the present state of the cube. Pick the blocks that are going to be used in the microfluidics. In this case, we numbered the selected blocks from 1 to 7. Blocks 1 and 7 are inlets/outlets blocks, blocks 2 and 6 are straight channels, blocks 3 and 5 are turnings, and block 4 is a spiral channel. **b** In the Rubik’s solver, generate an unscrambled cube, and then designate the position of each block that it will appear in the final arrangement. Record the colors of each block. **c** Reset the Rubik’s solver, and then paint the present positions of the useful blocks with their final colors. **d** Randomly paint the remaining unused blocks with legal colors on each block. **e** Click solve to calculate the algorithm. This process is usually done in a few seconds. An algorithm will be shown along with the rotation diagram of solving the cube. **f** If the program shows an invalid scramble, then follow the instructions to adjust the unused blocks to make it solvable. **g** Apply the given algorithm to the microfluidic cube. One will achieve desired microfluidic configuration after the final rotation

Now that we know most of the microfluidic combinations that are achievable by twisting the cube, we are curious about how easy it is to reconfigure the microfluidic cube. If we regard the state of the desired configuration as the unscrambled state of the cube, then the process of configuring the microfluidic cube is exactly the process of solving an ordinary Rubik’s cube, which can be done using either basic or advanced methods. A skilled player can solve a scrambled Rubik’s cube in a minute, and professionals could do this in just a few seconds. However, in a situation where users are not familiar with Rubik’s cube algorithms and the desired configuration is complex, the reconfiguration process could be a difficult task. Luckily, with the help of highly developed computing power, the search for an optimized solution of Rubik’s cube can be done with a computer program in just a couple of seconds. Similarly, we can use a computer program to help in reconfiguring the microfluidic cube. Figure 5 shows the process of reconfiguring the microfluidics with the help of an online Rubik’s cube solver^31^. By setting the final arrangement of microfluidic blocks as the unscrambled state, an algorithm for configuration could be calculated. It is noteworthy that the calculated algorithm is a relatively optimized solution to Rubik’s cube, but not necessarily the easiest way to reconfigure the microfluidics, as unused blocks that do not need to be restored are also turned to designated positions. In the future, we would like to design a program to restore specific blocks of the cube so that the calculation of the algorithm and reconfiguration of microfluidics could be more efficient. It has been proven that the maximum number of moves required to restore any of the permutations of Rubik’s cube, the so-called “God’s number,” is 20^32^. This conclusion can also be applied to the microfluidic cube; that is, if we wish to configure a specific microfluidic system from a completely unarranged state, then a total of 20 moves should be sufficient.

### Applications of the microfluidic cube

**Fig. 6:**
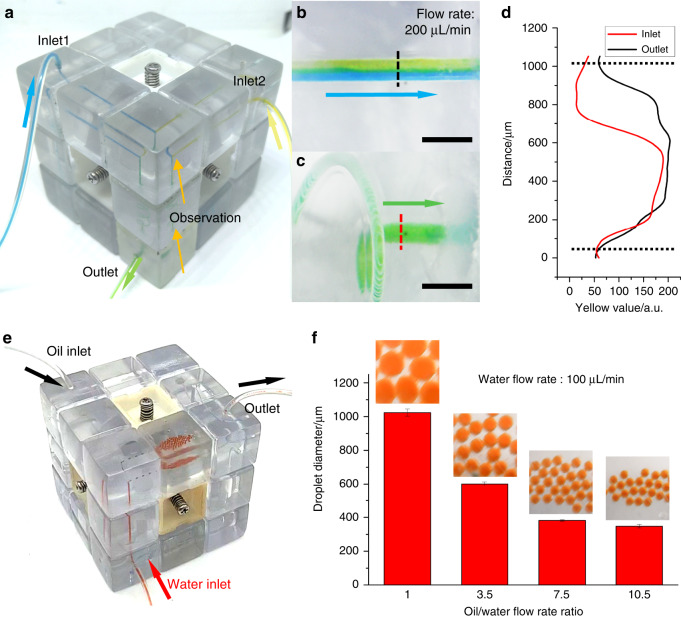
Photographs of functional microfluidics systems configured using the proposed cube. **a** A microfluidic mixer consisting of the inlet/outlet, channel, junction, and spiral channel blocks. Enlarged images of dye solution flow before entering (**b**) and leaving (**c**) the spiral channel block (scale bars: 2 mm). **d** Yellow value distribution in the microfluidic channel with changing distance along the dashed lines. **e** A water-in-oil droplet generator consisting of the inlet/outlet, channel, junction, and planar chamber blocks. **f** Droplet size variance when changing the water/oil flow rate ratio

The proposed microfluidic cube has several advantages over previously reported modular microfluidics, such as leakproof, ease of use, and disassembly free reconfiguration. To demonstrate the usefulness of the proposed microfluidic cube, Fig. 6 shows some examples of the configurations of the cube for different microfluidic application scenarios. Mixing is a basic microfluidic function that is used in all kinds of microfluidic devices^33^. Figure 6a shows the microfluidic cube configured for sample mixing that utilizes seven cube blocks: three inlet/outlet blocks, a T-junction block, two straight channel blocks, and a spiral channel block. When the blue and yellow dye solutions are infused from the inlets, the two flows converge in the T-junction block with an apparent boundary between them due to laminar flow (Fig. 6b). After flowing through a narrow spiral channel block, the boundary disappears, and the liquid becomes a homogeneous green color, as shown in Fig. 6c, indicating that the two dyes have mixed thoroughly. We further analyzed the color distribution at the dashed lines of in/outlets through image processing. The images of the in/outlets are converted to the CMYK color space, and the yellow channel value is used to represent the color variance in the channel. Figure 6d shows the yellow distribution along the dashed line, from which we learn that after flowing through a spiral mixer, the unbalanced color distribution turns to a relatively uniform color. The result also shows a decrease in the yellow value at the edge of the channel in both the inlet and outlet, which is caused by the thinning of the liquid at the edge of round channels.

Afterwards, we would like to reconfigure the microfluidic cube to a droplet generator. The configuration of the droplet generator is shown in Fig. 6e, which also utilizes seven cube blocks: three inlet/outlet blocks, a T-junction block, a chamber block, and two straight channel blocks. In the demonstration, water-in-oil droplets are generated in the T-junction block and then are collected in the chamber block for observation and further operations such as incubation. By fixing the flow rate of the water phase (100 μL/min) and tuning the flow rate of the oil phase (from 100 to 1050 μL/min), droplets with diameters ranging from 348 to 1023 μm could be generated, as shown in Fig. 6f. A supplementary movie for droplet generation is also available (Supplementary Movie S3). The droplets generated have uniform size and could be considered monodispersed. Such droplet microfluidic devices enable large amounts of parallel reactions and are useful in many applications that require high throughput, for example, digital nucleic acid amplification^34^ and cell screening^35^.

**Fig. 7:**
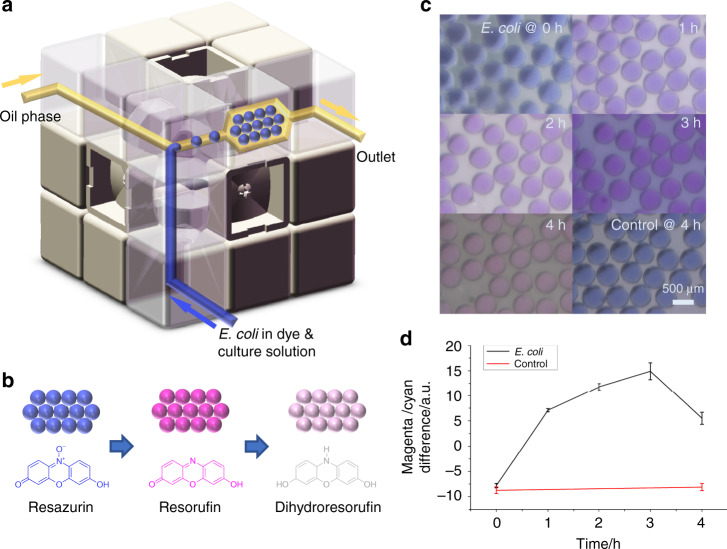
Droplet-based bacterial cell culture in the microfluidic cube. **a** Experimental setup of the microfluidic cube for a droplet-based bacterial culture. **b** Mechanism of the color change with resazurin reduction in the droplets. **c** Images of the droplets with varying time of incubation. **d** Estimated resorufin concentration in the droplets at different incubation times

As a demonstration of real application, we conducted a droplet-based microbial culturing experiment using the proposed microfluidic cube. Microbial culturing is essential for many subjects, such as diagnostics, genetics, and bioengineering. The culturing of bacteria in microscale droplets could allow the generation, manipulation, and monitoring of small populations of bacteria in a highly parallel and high-throughput manner, which could create new approaches for solving problems in diagnostics and for research on bacterial evolution^36^. In our experiment, *Escherichia coli* culture was added to Luria-Bertani (LB) media containing kanamycin and resazurin and was used as the water phase. For the oil phase, liquid paraffin containing an EM90 surfactant was used to encapsulate the droplets. The system setup for the experiment is shown in Fig. 7a, and the details are described in the Materials and methods section. After adding the *E. coli*, the culturing media were immediately dispersed to droplets and collected in a plenary chamber. Then, the microfluidic cube was incubated at room temperature (25 °C). The droplets in the chamber were observed over time. Since resazurin is a widely used cell viability indicator, the color of the droplet should change with bacterial growth. The cell activity could reduce the resazurin to highly fluorescent resorufin, changing the color of the droplet from blue to pink, as illustrated in Fig. 7b. As the reaction continues, the bacterial cells will further reduce the resorufin to nonfluorescent, colorless dihydroresorufin, and the droplet color could fade. As a result, we could monitor the cell activity through the color change of the droplets. Figure 7c shows the photographs of the droplets in the chamber during incubation. It can be inferred that the droplet color turned from blue to pink at first and faded afterwards, proving the existence of bacterial activity in the droplets. As a control, LB media containing resazurin and kanamycin were directly dispersed to droplets in the cube configuration without adding *E. coli*. The droplet color did not obviously change after 4 h of incubation. Due to the color difference between resazurin and resorufin, we could estimate the concentration of resorufin in the droplets through image processing. Figure 7d shows the estimation of resorufin concentration by calculating the magenta/cyan value difference at the center of droplets, as the color of resazurin and resorufin is close to the standard blue and magenta color, while blue is an equal mix of cyan and magenta. The result in Fig. 7d shows that the estimated resorufin concentration in the droplets increased at first but decreased after 3 h, which corresponds well to our explanation.

## Discussion

In conclusion, we present a novel method for rapidly building custom microfluidic systems by playing a microfluidic Rubik’s cube. Building with specially designed edge and corner blocks with internal microstructures, the microfluidic cube allows the flexible assembly of different microfluidic blocks by simply rotating the faces of the cube. After each rotation, all the blocks are self-aligned and sealed. Moreover, users can design and realize versatile microfluidic functions under the guidance of Rubik’s cube algorithms. As a proof of concept, we successfully created the proposed cube using 3D-printed blocks and configured it into functional microfluidic systems such as a mixer and a droplet generator. We also conducted a droplet-based bacterial cell culture using the microfluidic cube to demonstrate the usefulness of the proposed method in real applications. The cube-based microfluidic systems exhibited good reconfigurability and robustness and are therefore suitable for applications requiring rapid on-site deployment.

In the future, we would like to further expand Rubik’s cube-based microfluidic system to more applications by means of integrating versatile functional blocks with reference to previously reported strategies. For instance, we could build thermal regulation blocks for in-cube control for various reactions^37–42^; we could also build miniature pump and valve blocks^43^ for self-sufficient actuation of fluid flow. Sensing blocks with various kinds of sensors, such as electrochemical sensors^44^ and fiber-optic sensors^45^, shall also be integrated for in-cube detection and analysis. Apart from 3D printing, other prompt fabrication methods^46,47^ could also be adapted for more flexible design of the cube components. We also plan to expand the microfluidic cube to higher-order cubes (4 × 4 × 4 or 5 × 5 × 5) for more versatile combinations and to develop dedicated smartphone software for calculating optimized reconfiguration algorithms. Appealing in its convenience, performance, and ease of use, we hope the accomplishment of this work could help researchers from different fields who have limited knowledge in microfluidics to promptly deploy custom microfluidic systems in resource-limited settings.

## Materials and methods

The fabrication of Rubik’s microfluidic cube is a five-step process: designing, printing, washing, finishing, and assembly, which are described from section “Describing” to section “Assembly.” Section “Droplet-based *E. coli* culturing” describes the materials and methods used for droplet-based *E. coli* culturing.

### Designing

The microfluidic cube is adapted from Rubik’s original design. The cube has a total of 27 plastic components: 12 edge blocks, 8 corner blocks, 6 central blocks, and 1 cube core. The cube blocks have appearances that are similar to ordinary Rubik’s cube components, but have microstructures inside. The in/outlets are located at the center of the faces that contact adjacent blocks. Round channels with varying diameters ranging from 500 to 1500 μm are set in the blocks, giving various structures such as such as helices, chambers, and T-junctions. The detailed dimensions for the cube blocks are shown in Supplementary Fig. S4. All cube blocks are drawn in Solidworks and output to an STL format.

### Printing

The printing of the cube components is completed using a desktop SLA printer (Form2, Formlabs, USA). Clear resin V4 (RS-F2-GPCL-04) is printed with a 100 μm layer thickness. The supports are generated on the outer surfaces of each edge and corner block using default settings in the PreForm software. Before printing, it is important to check that no support is added to the O-ring concave (Supplementary Fig. S5) to ensure that the O-ring could conformally fit into the concave surface after the supports’ removal.

In this work, we use a minimum channel width of 500 μm for easier washing out of the uncured resin, although similar hardware and resin could allow a minimum void size of 220 μm, as demonstrated in previous studies^48^. Moreover, state-of-the-art 3D-printed microfluidics using desktop photopolymerization printers have a minimum void size of 20 μm × 18 μm^13^. Although such systems and resins are not commercially available yet, it is likely that we will be able to print cube components with significantly improved details in the near future.

### Washing

After printing, the printed components are put into an IPA (isopropyl alcohol) bath immediately, and the edge/corner blocks are evacuated by injecting IPA into the printed blocks using a syringe. Afterwards, the components are ultrasonicated for 10 min in the IPA container. Then, all the components are removed and dried for 12 h.

### Finishing

For clear observation, the edge and corner blocks are polished on out facelets. To do this, wetted abrasive papers (Stacke GmbH, Germany) are used to polish these facelets. Gradually, the grits of the abrasive paper (1000–7000) are increased during the finishing process until the block becomes transparent and the void can be clearly observed from outside. Afterwards, the polished blocks are washed with deionized water.

### Assembly

Silicone rubber O-rings are embedded in the two concaves of each edge block. Then, all components are assembled by assembling an ordinary Rubik’s cube where screws of M3 × 20 mm and springs of 0.6 mm (wire diameter) × 6 mm (outer diameter) × 15 mm (length) are used. Before assembly, M3 threads are tapped in all six holes of the cube core using a manual threading tap.

### Droplet-based *E. coli* culturing

*Escherichia coli* BL21-pET28a-egfp, a kind gift from the Central Laboratory of the Logistics University of Chinese People’s Armed Police Force, was used for the droplet-based bacterial culture. *E. coli* was prepared by culturing for 16 h in LB media (Solarbio Science & Technology Co., Ltd., Beijing, China) in a 37 °C, 180 r.p.m. incubator shaker before use. One hundred microliters of the *E. coli* culture was added to 5 ml LB media containing resorufin (150 μg/mL) and kanamycin (50 μg/mL). The centrifuge tube was then shaken to mix the *E. coli* with LB media. Before generating droplets, the microchannels of the microfluidic cube were rinsed by injecting medicinal alcohol for 10 min. LB media containing *E. coli* was used as the water phase (flow rate 50 μL/min), and liquid paraffin (Kermel Chemical Reagent Co., Ltd., Tianjin, China) with 3% EM90 (Evonik, Germany) was used as the oil phase (flow rate 100 μL/min) to generate droplets. After droplet generation, the microfluidic cube was placed in a 25 °C environment for incubation. Photographs of the droplets were taken over 4 h using a stereomicroscope (SMZ18, Nikon, Japan) and camera (DP21, Olympus, Japan). To estimate the relative concentration of resorufin in droplets, the photographs of the droplet chamber are converted to the CMYK color space, and the difference between the magenta and cyan channels at the central 25 pixels of each droplet is used as an estimation of the resorufin concentration. For each image, the estimation is calculated for 10 droplets.

## Supplementary information

Supplementary Movie S1: Replacing an Edge Block

Supplementary Movie S2: Replacing a Corner Block

Supplementary Movie S3: Water-in-oil Droplet Generation

Supplementary Information
