# Supplementary material for: A Rubik’s microfluidic cube
Source: Microsyst Nanoeng. 2020 Jun 15;6:27. doi: 10.1038/s41378-020-0136-4 (PMC8433458; doi:10.1038/s41378-020-0136-4)
Supplement: Supplementary file 4 — Supplementary Information [file 41378_2020_136_MOESM4_ESM.pdf]

Supplementary Information

A Rubik's Microfluidic Cube

*Xiaochen Lai[a], Zhi Shi[a], Zhihua Pu[a], Penghao Zhang[a], Xingguo Zhang[a], Haixia Yu[b], Dachao Li[a]**

[a] State Key Laboratory of Precision Measurement Technology and Instruments, Tianjin University, Tianjin, 300072, China
*E-mail: dchli@tju.edu.cn

[b] Tianjin Key Laboratory of Biomedical Detecting Techniques and Instruments, Tianjin University, Tianjin, 300072, China

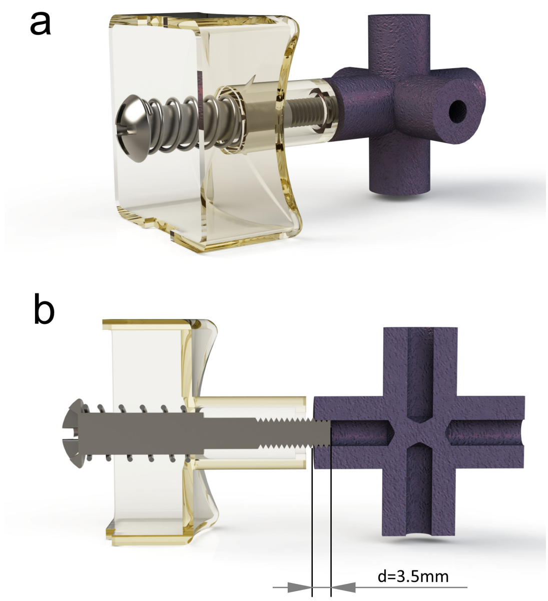

Figure S1. a. Illustration about how a central block is fixed to the cube block with a screw and a spring. **b.** Section wiew of **a** where the depth of the screw driven into the cube is 3.5 mm, ensuring smooth rotation and leakproof connection of microfluidic cube blocks.

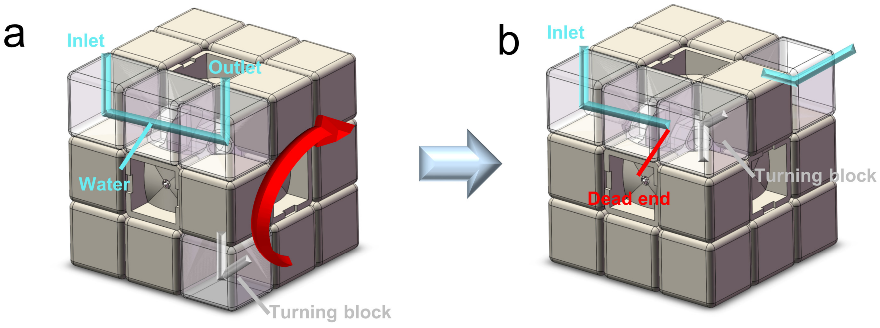

Figure S2. Experimental setup of the pressure resistance test of the microfluidic cube. **a.** At first, The microfluidic cube is configured to have 3 blocks: Inlet, straight channel and outlet. Water are injected into the cube to fill the channel. **b.** Afterwards, turn a turning corner block to replace the outlet block to form a dead end for the microchannel. Then, pressure generated by an air compressor and a pressure relief valve is applied to the inlet to verify the pressure resistance.

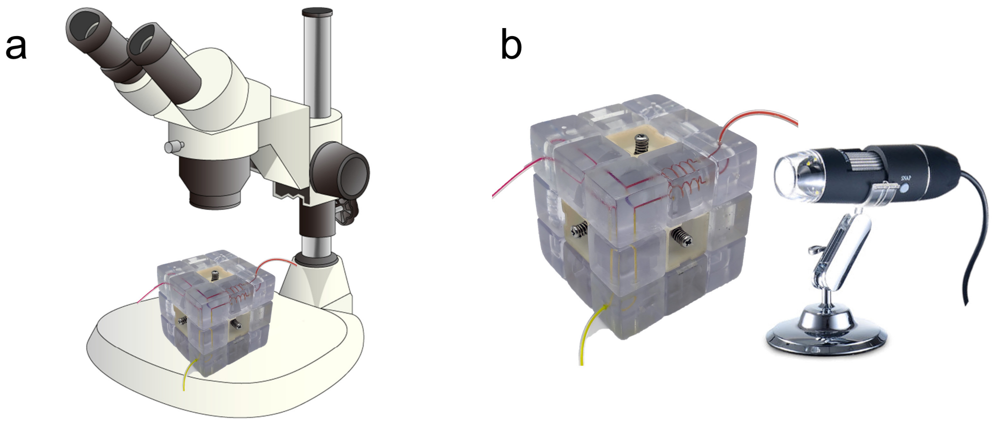

Figure S3. Illustration of current strategies to observe the microchannels in the microfluidic cube. **a.** Observe the top surface of the cube with a stereomicroscope. **b.** Observe the side surface of the cube with a desktop usb magnifier.

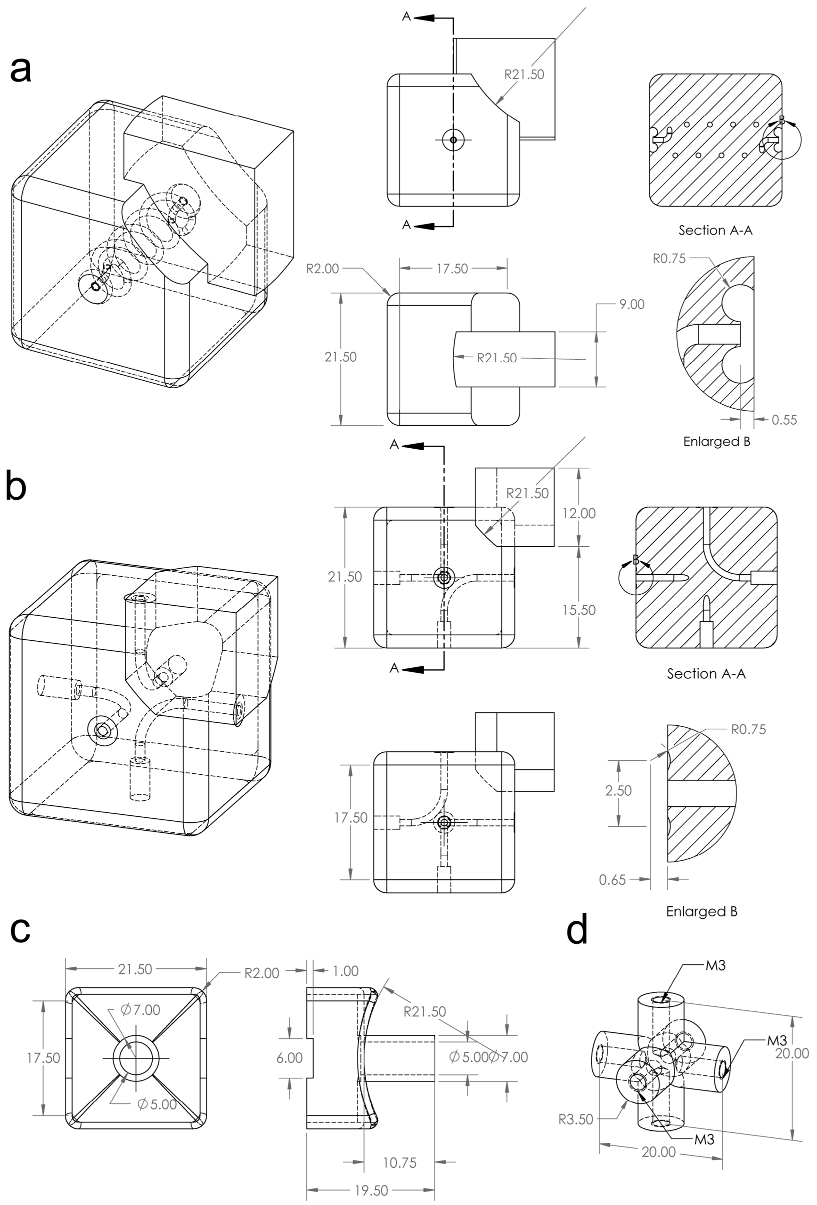

Figure S4 Designed dimensions of the components of the microfluidic cube. **a.** An edge block with a spiral channel. Right bottom: enlarged view of the O-ring embedding torus concave. **b.** A corner block of a 3-way inlets/outlets. Right bottom: enlarged view of the O-ring fitting torus concave. **c.** A central block. **d.**The cube core. All values are in mm.

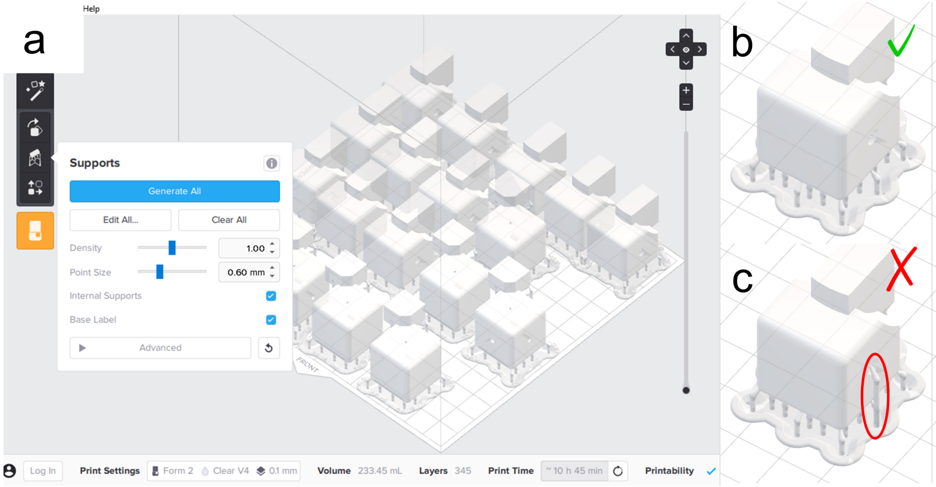

Figure. S5 Adding supports for 3D printing. **a.** Orientation of edge and corner blocks. All blocks are oriented with an out face connecting the supports so that the models are printable without adding additional support to the O-ring convex. **b.** A right example of the supports. **c.** A wrong example of the supports. The support at the convex (in the red ellipse) will cause a lump in the convex after removal. In this case the O-ring will be unable to completely fit into the convex.
